# 

**Published:** 1879-06

**Authors:** 


### CONTENTS:

ORIGINAL COMMUNICATIONS:

I. On the Conduct of the Third Stage of Labor. By DeLaskie Miller, M.D....... 562

II. A Lesson from Ovariotomies. By J. T. Everett, M.D........................... 568

III. Of Miscarriage, and Especially of the Use of Loomis' Forceps in its Management. By Henry A. Martin, M.D.. 578

IV. A Case of Unusually Late Chloroform Ashhyxia. By F. C. Hotz, M.D... 591

V. Antiseptic Surgery. By G. B. Pratt, M.D............................................ 594

CLINICAL REPORTS:

VI. Chicago Hospital for Women and Children............................................ 600

*Notes from Private Practice:*

VII. Case of Gangrene of the Leg from Phlegmasia Dolens; Case of Tracheotomy for Tumor at the Glottis............................................ 605

SOCIETY REPORTS:

VIII. Transactions of the Chicago Gynæcological Society.......................... 611

IX. The Brainard District Medical Society............................................ 643

X. State Microscopical Society............ 614

XI. Michigan State Board of Health... 615

TRANSLATIONS:

Prof. Lefort on the Antiseptic Treatment of Wounds........................................ 623

SELECTIONS:

XIII. Report on Histology and Microscopy........................................ 627

FOREIGN CORRESPONDENCE:

XIV. London Letter. By C. T. Parkes, M.D........................................ 635

XV. Emmet's Operation. By R. Barnes, M.D........................................ 643

DOMESTIC CORRESPONDENCE:

XVI. New York Letter........................ 644

XVII. Index Medicus. By R. Fletcher, M.D........................................ 647

REVIEWS AND BOOK NOTICES.................... 650

Books and Pamphlets Received............. 661

OBITUARY:

John M. Woodworth, M.D..................... 664

ITEMS............610, 622, 626, 634, 649, 663, 671

ANNOUNCEMENTS FOR THE MONTH............ 672

THE METRIC SYSTEM............. xix, xxii advts.

Notice to Contributors..................... xx advts.

